# A time-course Raman spectroscopic analysis of spontaneous in vitro microcalcifications in a breast cancer cell line

**DOI:** 10.1038/s41374-021-00619-0

**Published:** 2021-06-11

**Authors:** Pascaline Bouzy, Shane O’Grady, Honey Madupalli, Mary Tecklenburg, Keith Rogers, Francesca Palombo, Maria P. Morgan, Nicholas Stone

**Affiliations:** 1 School of Physics and Astronomy, University of Exeter, Exeter, UK; 2 School of Pharmacy and Biomolecular Science, Royal College of Surgeons in Ireland, Dublin, 2, Ireland; 3 Department of Chemistry and Biochemistry and Science of Advanced Materials Program, Central Michigan University, Mt. Pleasant, MI USA; 4 Cranfield Forensic Institute, Cranfield University, Shrivenham, UK

**Keywords:** Breast cancer, Cancer models

## Abstract

Microcalcifications are early markers of breast cancer and can provide valuable prognostic information to support clinical decision-making. Current detection of calcifications in breast tissue is based on X-ray mammography, which involves the use of ionizing radiation with potentially detrimental effects, or MRI scans, which have limited spatial resolution. Additionally, these techniques are not capable of discriminating between microcalcifications from benign and malignant lesions. Several studies show that vibrational spectroscopic techniques are capable of discriminating and classifying breast lesions, with a pathology grade based on the chemical composition of the microcalcifications. However, the occurrence of microcalcifications in the breast and the underlying mineralization process are still not fully understood. Using a previously established model of in vitro mineralization, the MDA-MB-231 human breast cancer cell line was induced using two osteogenic agents, inorganic phosphate (Pi) and β-glycerophosphate (βG), and direct monitoring of the mineralization process was conducted using Raman micro-spectroscopy. MDA-MB-231 cells cultured in a medium supplemented with Pi presented more rapid mineralization (by day 3) than cells exposed to βG (by day 11). A redshift of the phosphate stretching peak for cells supplemented with βG revealed the presence of different precursor phases (octacalcium phosphate) during apatite crystal formation. These results demonstrate that Raman micro-spectroscopy is a powerful tool for nondestructive analysis of mineral species and can provide valuable information for evaluating mineralization dynamics and any associated breast cancer progression, if utilized in pathological samples.

## Abstract (web summary)

The mineralization of MDA-MB-231 breast cells was investigated using Raman micro-spectroscopy. Mineralization was induced by two osteogenic agents: inorganic phosphate (Pi) and β-Glycerophosphate (βG). The results show that the uptake of Pi allows a faster mineralization and the uptake of βG indicated the presence of a precursor phase during the hydroxyapatite crystal formation.

## Introduction

In 2018, breast cancer was one of the most commonly diagnosed types of cancer and the second cause of death from cancer worldwide (after lung cancer) [1]. Around 2.1 million women across the world were diagnosed with breast cancer in 2018 [1] with survival rates which are predicted to increase over the next decades, mainly due to changes in detection practice by screening individuals more often and earlier [2]. Early-stage diagnosis is important to improve the survival rate and treatment response, and in this context microcalcifications appear to be the most valuable marker of breast cancer [3]. X-ray and vibrational spectroscopy techniques are widely used to investigate the composition of these crystals [4–9]. For instance, it has already been shown that type I microcalcifications are composed of calcium oxalate dihydrate and are observed only in benign lesions, whilst type II microcalcifications are mainly composed of calcium hydroxyapatite (Hap) and are associated with both proliferative benign lesions and malignant lesions [10]. Moreover, in biological tissue, the Hap crystal is not found in its stoichiometric form as the lattice contains carbonates (CO_3_^2−^ ions) which reduce the stability of the crystal and increase its solubility [11–13] with detrimental effects. Two types of substitution are found in the Hap lattice: type A, in which carbonate replaces a hydroxyl ion (OH^−^), and type B, where carbonate replaces a phosphate ion (PO_4_^3−^) [14]. Baker et al. have previously demonstrated using FTIR micro-spectroscopy that the extent of carbonate substitution within breast microcalcifications directly correlates with the degree of pathology [5], a finding that is in line with results of other Raman studies [4, 5, 15]. However, further analysis shows that the calcification’s chemical composition is more complex and suggests the presence of a close interplay between microcalcifications and their microenvironments, potentially affecting both formation and maturation of the crystals.

It is known that the regulation of tumor pH plays an important role in cell proliferation and cancer progression. Several studies have shown that cells within a tumor experience hypoxia and upregulation of metabolic processes. Changes in metabolic activity lead to an increase of intracellular concentrations of H^+^ hydrogen ions and carbon dioxide, which are then released (via different transporters) into the microenvironment causing a decrease of pH [16] and promoting breast cancer progression [17]. The acidic tumor microenvironment can give rise to different precursors in microcalcifications [18]. In addition, recent studies suggest that microcalcifications may contain other mineral phases such as magnesium-substituted β-tricalcium phosphate (β-TCP or whitlockite) [15, 19].

In this study, we hypothesized that, in addition to the calcium Hap, different phosphate species as mineral precursors (e.g. octacalcium phosphate (OCP) or amorphous calcium phosphate (ACP)) could be found in the calcium deposits [20].

To gain an insight into the crystal formation process, we evaluated two different pathways of cell mineralization using a model developed and validated in several studies by Morgan et al. [3, 21]. The first pathway consists of using an osteogenic cocktail (OC) composed of β-glycerophosphate (βG), which is a source of inorganic phosphate, and ascorbic acid (AA), which promotes collagen production, with added dexamethasone (Dex), which is considered as an inducer of osteoblastic differentiation. The second pathway uses inorganic phosphate (Pi) combined with Dex, which induces faster mineralization [21].

Here, we report a mineralization of the MDA-MB-231 breast cell line after 3 days when the cells were cultured with Pi. The Raman spectroscopic analysis demonstrated a heterogeneity of microcalcifications in terms of mineral species and also protein level. In fact, the mineralization process involved different phosphate species i.e. OCP and β-TCP during Hap crystal formation. In parallel, variations of protein and DNA peak intensities and an increase of the mineral-to-matrix ratio (MMR) were also observed during the cell mineralization.

The aim of this study was to use an established cellular model of mammary microcalcification to characterize the mineralization pathway of the breast cancer cells using Raman spectroscopy.

## Methods

### Induction of cell mineralization

**Fig. 1:**
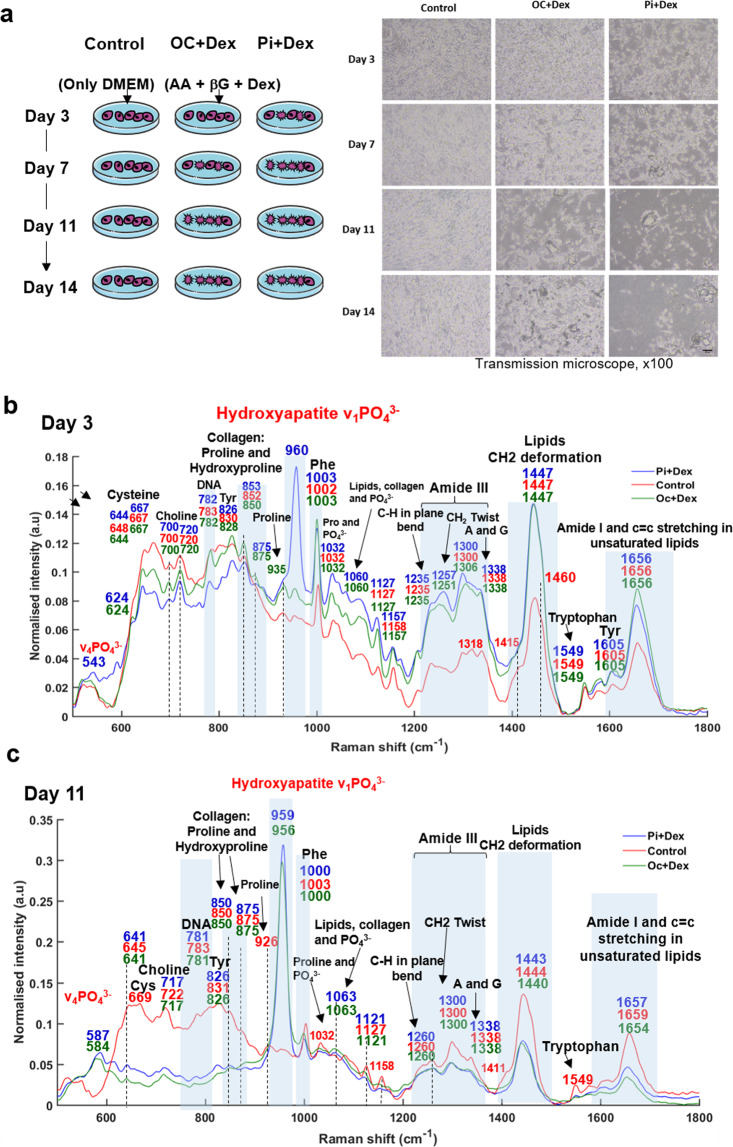
Experimental design and specific Raman features of mineralization. **a** Protocol used for 14-days mineralization of MDA-MB-231 cells (left panel). Acronyms denote OC osteogenic cocktail, Dex dexamethasone, Pi inorganic phosphate, DMEM Dulbecco’s modified Eagle medium, βG β-glycerophosphate, AA ascorbic acid. Representative images of MDA-MB-231 cells growing at different time point of mineralization (Day 3, 7, 11, and 14) (right panel). Scale bar: 20 µm and ×100 magnification. **b** Raman spectra acquired from mineral deposits in cell culture after 3 days and **c** 11 days of mineralization. Labels denote Phe Phenylalanine, Cys Cysteine, Tyr Tyrosine. Cells were treated with Pi + Dex (blue line) and OC + Dex (or βG) (green line). Non-treated cells were considered as control (red line). Each spectrum is an average of 40 spectra for each condition.

MDA-MB-231 cells were maintained in sterile 75 cm^2^ flasks with standard growth medium made of low glucose Dulbecco’s Modified Eagle Medium (DMEM, Thermofisher Scientific) supplemented with 10% fetal bovine serum (Thermofisher Scientific) and 1% penicillin/streptomycin (Thermofisher Scientific) at 37 °C and 5% CO_2_ atmosphere. After removing the medium, cells were subjected to treatment with trypsin (Thermofisher Scientific) to detatch them from the flask and washed with PBS. Cells were then seeded onto calcium fluoride (CaF_2_) slides (Crystran, Poole, UK) inserted in six-well plates at 0.8 × 10^6^ cells/well in 3 mL/well of medium and grown overnight. The following day (day 1), mineralization was induced by two pathways using osteogenic agents, as illustrated in Fig. 1a. Osteogenic reagents (10 mM β-glycerophosphate, 50 μg/mL ascorbic acid, and 100 nM dexamethasone; Sigma-Aldrich) were added to the cell culture medium in each well (OC + Dex). Alternatively, Pi (10 mM) and 100 nM dexamethasone was used (Pi + Dex) or cells were grown in normal media (control). Pi was prepared from Na_2_HPO_4_ and NaH_2_PO_4_ in 4:1 molar ratio as a 1 M stock solution. The solution was diluted in pyrogen-free water. The medium was refreshed every 3 days until the end of the study (day 14). The mineralization process was stopped at day 3, 7, 11 and 14. Cells were washed three times with PBS, fixed with 4% paraformaldehyde (Thermofisher Scientific) for 30 min and dried at room temperature. Duplicate slides were also analyzed following 3, 7, 11, and 14 days in culture using both IR and Raman microscopy.

### Mineral standards

B-type cHap powders were synthesized and provided by Prof. Mary Tecklenburg from Central Michigan University, USA. These standards were prepared by incorporating different contents of CO_3_^2−^ (1.24, 2.92, 4.43, 5.24, 7.52, and 8.12 wt.%) in the Hap lattice as part of the synthetic process. The preparation method used in this study was similar to those previously reported by Tecklenburg et al. [22, 23].

Another set of mineral standards comprising ACP, OCP, and β-TCP were prepared and provided by Prof. Keith Rogers from Cranfield University.

### Raman micro-spectroscopy

Micro-Raman measurements were acquired using a Renishaw inVia Raman microscope, comprising a ×50 long working distance objective (N.A. 0.5) for illumination and collection of the backscattered light, xyz motorized stage, 600 lines/mm grating, and deep-depletion CCD camera. The excitation source was a near infrared diode laser with 830 nm wavelength.

For each mineral standard in powder form, ten single-point spectra were recorded at different locations with 5 s acquisition time and 5 accumulations/spectrum using WiRE software. Two replicates (2 × 10 spectra) were performed, and an average spectrum was obtained.

Raman 2-D maps of mineralized cells at specific days of mineralization (day 3, 7, 11, and 14) were recorded in streamline mode with 30 s acquisition time, 9 μm step size.

20 cells (*n* = 20) have been analyzed for each time point, each condition, each replicate and 2 spectra/cell were recorded. A total of 40 spectra were analyzed per condition, per day, per replicate.

### Data analysis

Datasets were analyzed using an in-house Matlab code (Mathworks, USA). In Raman maps, spectra of mineralized cells were selected and extracted from a central location within the mineral deposit based on the phosphate peak intensity at 960 cm^−1^. An average spectrum was calculated from 10 spectra of mineral standards and 40 spectra of mineralized cells for each replicate. Raman spectra were truncated in the spectral regions of interest for the Amide I (1710–1615 cm^−1^), CH_2_ deformation (1500–1400 cm^−1^), Amide III (1380–1215 cm^−1^), carbonate (1100–1010 cm^−1^), and phosphate stretching (985–900 cm^−1^) bands. Raman spectra were pre-processed as follows: a smoothing was performed using the Savitzky-Golay algorithm, second order polynomial with 9 points (OPUS software, Bruker Optics, Ettlingen, Germany). Then, a baseline correction using the rubber-band method with 64 data points was also performed on each mean spectrum (OPUS software, Bruker Optics, Ettlingen, Germany). Second derivative spectra were calculated by using a Savitsky–Golay algorithm with nine points smoothing to identify subband positions to use in the curve fitting analysis.

Curve fitting analysis of spectral band shapes was performed using OriginPro software (OriginLab, USA). A linear combination of Gaussian and Lorentzian curves (pseudo-Voigt function) was used to fit the carbonate and phosphate bands. The Levenberg–Marquardt algorithm was used to minimize the χ^2^ value. The peak positions selected from the second derivative spectra were fixed whilst the linewidths were free fitting parameters.

Statistical analysis was performed using GraphPad Prism 6 software (La Jolla, CA, USA). To evaluate the involvement of mineral phases within the microcalcifications, a two-way ANOVA test was applied to the different treatments and time points.

Principal component analysis (PCA) was performed on the Raman maps of mineralized cells and also on the average spectra extracted from the maps for the different conditions using a toolbox in Matlab software. Prior to the PCA, a cosmic ray removal was performed using a median filter, followed by a baseline correction with asymmetric least square smoothing and a vector normalization in Matlab.

## Results

### Time-course of the mineralization

One of the advantages of using Raman micro-spectroscopy is that the changes induced by mineralization are readily identified without the need for a specific label or histological staining and without damage to the sample. Figure 1b shows Raman spectra extracted from cellular mineral deposits after 3 days of cell culture with osteogenic agents, compared with control cells. The spectrum of cells cultured with Pi exhibit a distinct peak at around 960 cm^−1^ corresponding to the phosphate stretching (ν_1_ PO_4_^3−^) mode of cHap, indicating the onset of cell mineralization after only 3 days of treatment. For all conditions, we also found spectral features related to proteins (Amide I and III) and lipids at 1656, 1338, 1300, 1257, and 1447 cm^−1^ [24], collagen with hydroxyproline and proline signals at around 853, 875, and 935 cm^−1^ [25] and DNA at 782 cm^−1^ [20]. The same peak positions were found for cells treated with Pi at day 7 (Supplementary Fig. 1a).

Regarding cells treated with βG, the mineralization process is slower, beginning at day 11, as indicated by the phosphate peak at 956 cm^−1^ in Fig. 1c. This peak is slightly shifted compared to that at 959 cm^−1^ for cells treated with Pi. The same shifted position is observed for the phosphate band from cells treated with βG after 14 days of mineralization (Supplementary Fig. 1b). The peak position at 957 cm^−1^ plausibly suggests the presence of another mineral phase initially or a combination of different species with cHap crystal within the mineral deposits.

**Fig. 2:**
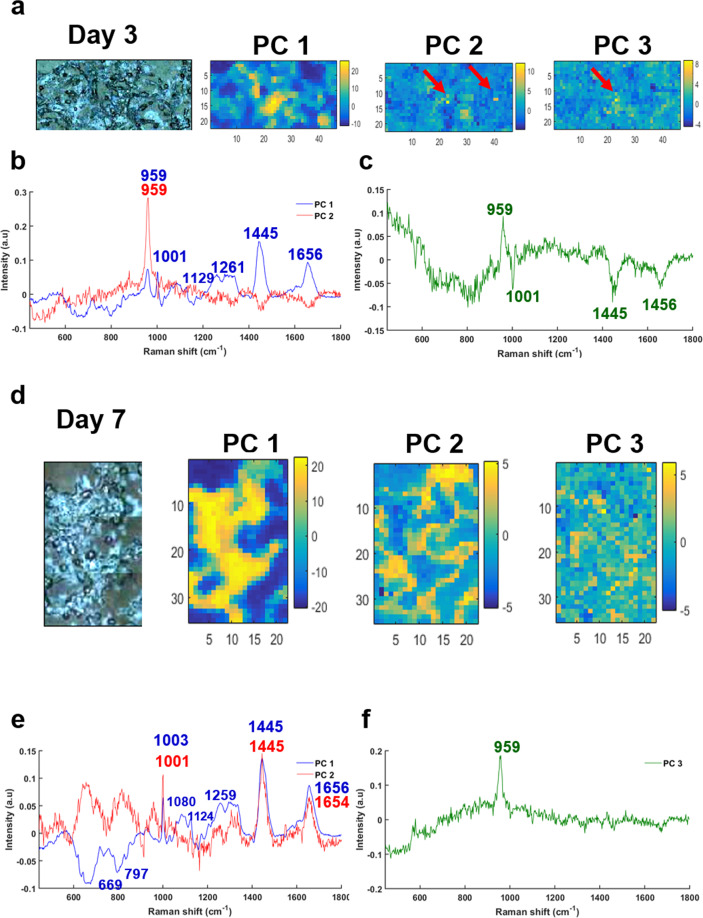
Results of PCA performed on a Raman map of breast cancer cells after 3 and 7 days of mineralization. **a** White light image of breast cancer cells treated with Pi at day 3 after mineralization and the first three PC scores, **b** corresponding PC1 and PC2 loadings and **c** PC3 loading. **d** White light image of breast cancer cells treated with Pi day 7 after mineralization and the first three PC scores, **e** corresponding PC1 and PC2 loadings and **f** PC3 loading.

To localize the sites of mineralization, a PCA was applied to all the Raman maps and, amongst the nine PCs computed in the analysis, the first three PCs were retained for comparison between samples. The maps from the early days of mineralization for cells cultured in a Pi-supplemented medium exhibit calcium deposits in the PC2 and PC3 scores at day 3 (in yellow, Fig. 2a) and PC3 score at day 7 (Fig. 2d). These deposits present the distinctive phosphate peak at 959 cm^−1^ in their PC loadings (Fig. 2b, c, f). For both days, PC1 loading denotes the cytoplasm of cells characterized by a combination of protein and lipid peaks at 1445, 1656, and 1260 cm^−1^ (Fig. 2b, e).

**Fig. 3:**
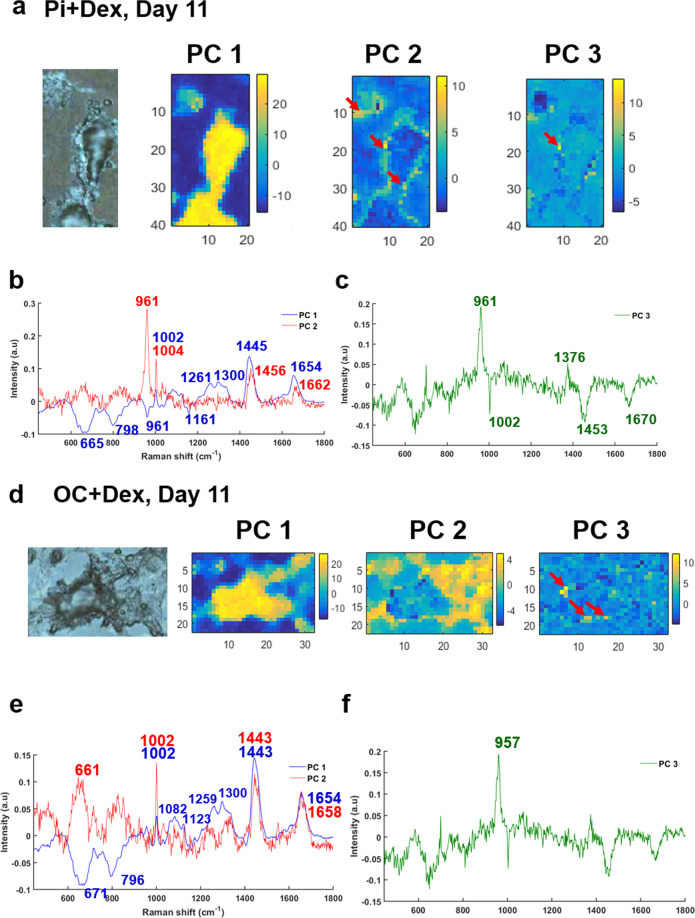
Results of PCA performed on a Raman map of breast cancer cells after 11 days of mineralization. Cells treated with (upper panel) Pi and (lower panel) βG. **a**, **d** White light images and the first three PC scores, **b**, **e** corresponding PC1 and PC2 loadings and **c**, **f** PC3 loadings.

At day 11, the PC3 score is still defined by the calcium deposit with yellow spots (red arrows, Fig. 3a) and corresponding loading plot showing a phosphate peak at 961 cm^−1^ (Fig. 3c), whilst the PC2 loading shows that cells contain a combination of cHap, proteins, and lipids at 1662 and 1445 cm^−1^ [26–28] (red line in Fig. 3b). The PC1 loading can be ascribed to the cell cytoplasm, with protein and lipid peaks (blue line in Fig. 3b).

**Fig. 4:**
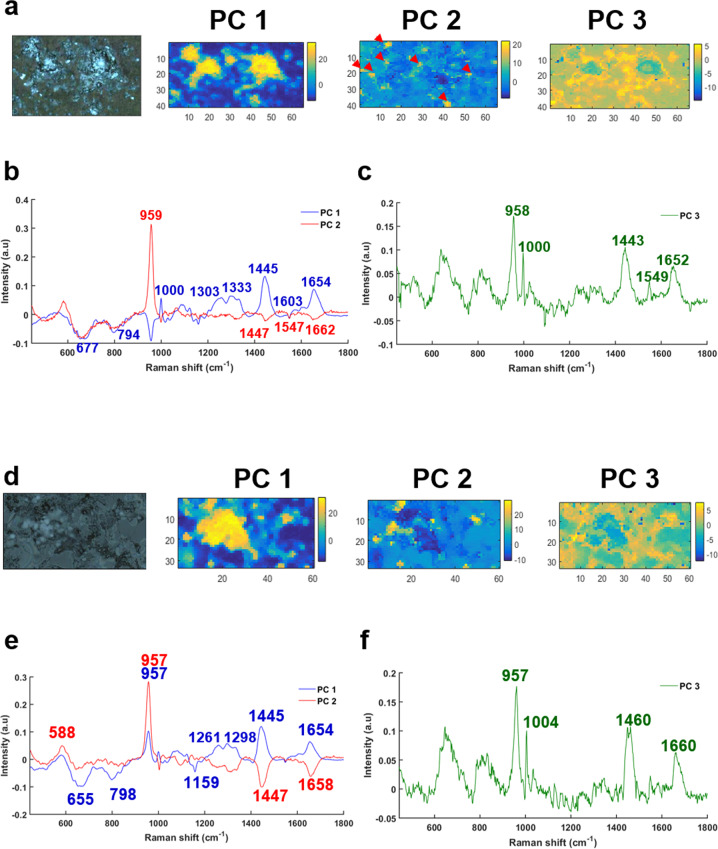
Results of PCA performed on a Raman map of breast cancer cells after 14 days of mineralization. Cells treated with (upper panel) Pi and (lower panel) βG. **a**, **d** White light images and the first three PC scores, **b**, **e** corresponding PC1 and PC2 loadings and **c**, **f** PC3 loadings.

Similar results were found for cells after 14 days of mineralization (Fig. 4), with the PC1 score representing the cell cytoplasm (yellow and orange, Fig. 4a) whilst the PC2 score denoting the calcium deposits with several small yellow spots (red arrows, Fig. 4a). The PC1 loading exhibits lipid and protein peaks at 1654, 1445, 1333, and 1303 cm^−1^ for Amide I, CH_2_ deformation and Amide III, respectively [26, 27] (Fig. 4b). The PC2 loading shows a single phosphate peak for Hap at 959 cm^−1^ corresponding to calcium deposits (Fig. 4b) without proteins or lipids. The PC3 loading, corresponding to the cytoplasm, exhibits the same phosphate peak at 959 cm^−1^ but also several peaks of proteins and lipids at 1443, 1549, and 1652 cm^−1^ (Fig. 4c).

The same analysis was performed on Raman maps from cells cultured in a medium supplemented with βG, after 11 and 14 days of mineralization. PC scores and corresponding loadings are shown in Figs. 3 and 4, respectively. At day 11, the PC3 loading exhibits a phosphate peak at 957 cm^−1^ which corresponds to the mineral deposits (Fig. 3f) and illustrated in yellow spots in Fig. 3d (red arrows), whilst the PC1 and PC2 loadings show several peaks for proteins and lipids (Amide I, CH_2_ deformation and Amide III) corresponding to the cytoplasm of the cells.

In the same way as the results obtained for the cells treated with Pi after 14 days of mineralization, for βG treatment the PC1 and PC3 loadings show a combination of proteins, lipids, and cHap bands (Fig. 4e, f). The calcium deposits are well-represented in yellow spots by the PC2 score (red arrows, Fig. 4d). These microcalcifications exhibit a phosphate peak at 957 cm^−1^ without any proteins or lipids (red line, Fig. 4e).

Moreover, by comparing phosphate peak positions, a shift is noticed between Pi and βG treatment. In fact, when cells are treated with Pi, the phosphate peak is around 959 cm^−1^ whilst when cells are treated with βG it is around 957 cm^−1^, which is in line with previous results and suggests the possible presence of another mineral [29].

**Fig. 5:**
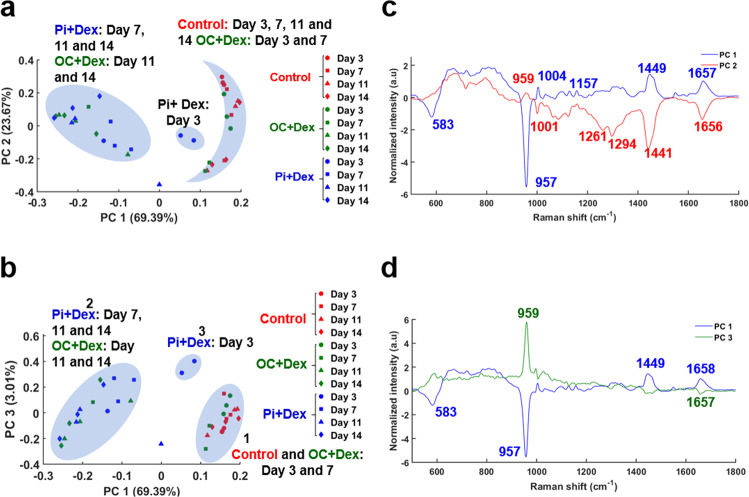
Time-course of the mineralization by PCA. Results of PCA performed on spectra that are average of 40 Raman spectra acquired from breast cancer cells at 3 days (dots), 7 days (squares), 11 days (triangles), and 14 days (diamonds) of mineralization and for each condition (control in red, cells treated with βG (OC + Dex) in green and Pi in blue). **a** PC1 and PC2 score plots and **c** corresponding loadings (blue and red); **b** PC1 and PC3 score plots and **d** corresponding loadings (blue and green).

To further investigate the onset of mineralization and the resulting chemical changes, PCA was performed on the average spectra extracted from breast cancer cells for each condition at day 3, 7, 11, and 14. PCA score and loading plots for the first three PCs are illustrated in Fig. 5. A PCA with PC1 and PC2 scores which represent 69.39 and 23.67% of the entire variance in the dataset can be divided in three groups or clusters (Fig. 5a). The first group is represented by control cells (all days) and cells treated with βG at day 3 and 7 (blue crescent-shaped). The second group contains the cells treated with Pi (at day 7, 11, and 14) and cells treated with βG at day 11 and 14 (blue ellipse). The main difference between these two clusters is in the presence of the phosphate peak in PC1 and PC2 loadings, specific for the mineral deposits. In fact, the cells treated with Pi have started their mineralization compared to the control group (Fig. 5c). The third cluster includes the cells treated with Pi at day 3 as they have started an early mineralization (Fig. 5a).

The PCA with PC1 and PC3 scores which represent 69.39 and 3.01% of the entire variance in the dataset can be divided in three clusters (Fig. 5b). As before, the first cluster regroups the control cells (all days) and cells treated with βG at day 3 and 7 (blue ellipse 1). The second cluster represents the cells treated with Pi (all days except for day 3) and cells treated with βG at day 11 and 14, as the cells have started their mineralization (blue ellipse 2). The difference between the two clusters is again in the phosphate peak in PC1 and PC3 loadings (Fig. 5d). The third cluster concerns the cells treated with Pi at an early phase of mineralization (day 3) (blue ellipse 3, Fig. 5b).

These results demonstrate different chemical changes during the mineralization process, in particular for the cells treated with Pi. In fact, two distinct clusters are highlighted: one group for the early mineralization at day 3 and another one for the other days (7, 11, and 14), which suggests that biochemical changes occur during the maturation of the mineral deposits. Moreover, the PC loadings (Fig. 5c, d) show different phosphate peak positions (959 and 957 cm^−1^), which tend to suggest that different phosphate species are present in the mineral deposits.

### Analysis of the spectral changes during the mineralization of breast cancer cells

To further analyze the mineral deposits and assess the biochemical changes during the maturation process, a peak decomposition was performed on the spectra extracted from cells at different days of mineralization and for each condition.

**Fig. 6:**
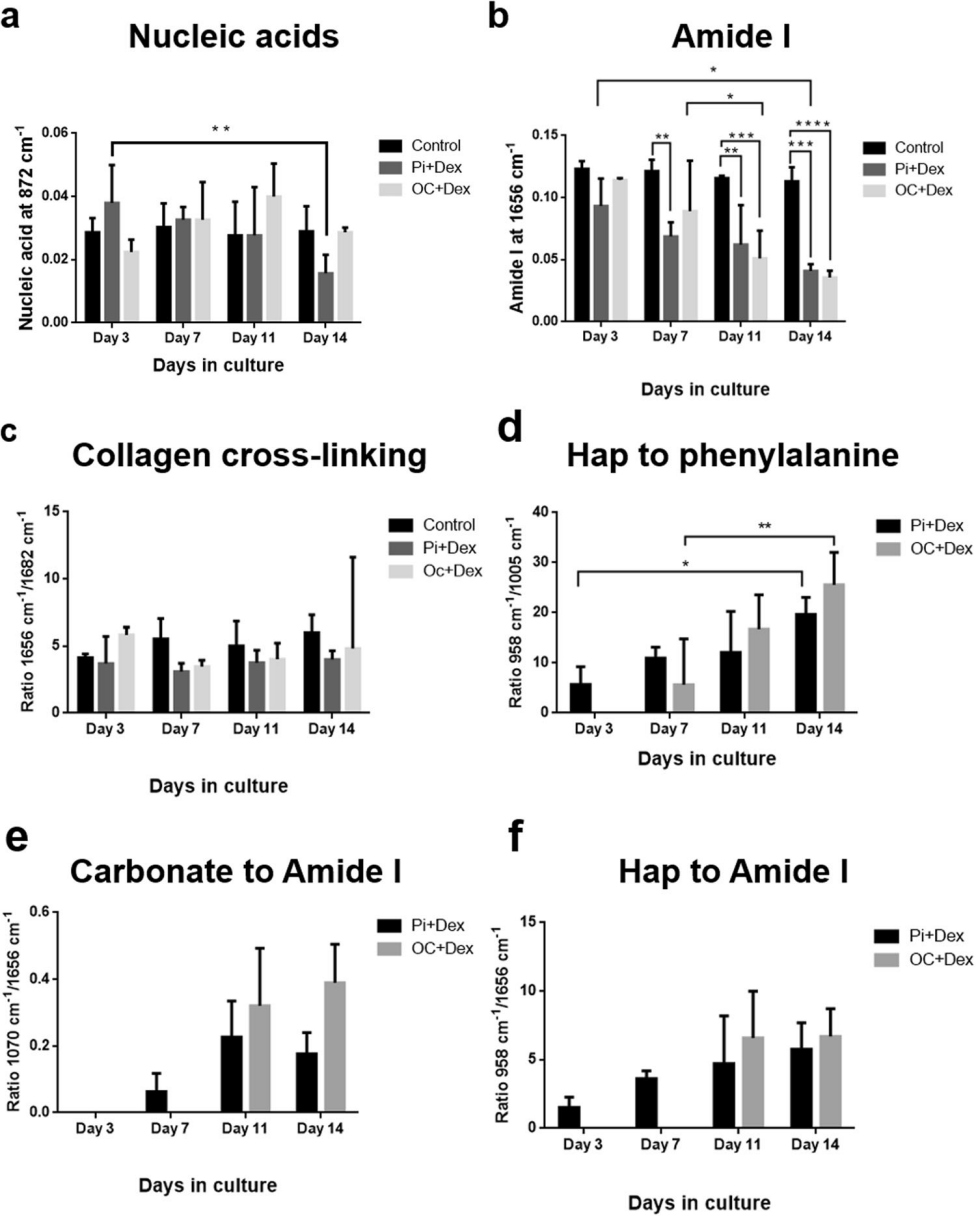
Assessment of Hap crystal quality. Evolution in peak intensity for **a** nucleic acid at 782 cm^−1^ and **b** Amide I at 1656 cm^−1^ for the control and the different treatments (OC + Dex and Pi + Dex) over time of mineralization. Two-way ANOVA test was performed for both: *P = 0.0318 and **P = 0.024 and ***P < 0.001, respectively. **c** Evolution in collagen cross-linking derived from the intensity ratio between Amide I (1656 cm^−1^) and the 1682 cm^−1^ peak obtained from the curve fitting, representing the maturation of collagen over time of mineralization. Evolution in mineral-to-matrix ratio (MMR) evaluated as the intensity ratio of **d** Hap (959 cm^−1^) to phenylalanine (1005 cm^−1^), **e** Hap (959 cm^−1^) to Amide I (1656 cm^−1^) and **f** carbonate (1070 cm^−1^) to Amide I (1656 cm^−1^) over time. Calculations were performed for the different treatments (OC + Dex and Pi + Dex). All data are presented as mean ± SD.

The peak intensities of DNA and Amide I (at 872 and 1656 cm^−1^, respectively) were compared for each day (Fig. 6). The analysis shows a high intensity and hence high concentration of DNA at day 3 for cells supplemented with Pi, followed by a significant decrease at day 14 (Fig. 6a). No significant changes are noted for the spectra extracted from cells treated with βG. Several studies tend to suggest that the cells may have stopped their proliferation phase to start the mineralization process [20, 30, 31]. In the same way, a significant decrease of proteins is observed from day 3 to 7 for cells treated with Pi and from day 7 to 11 for cells treated with βG (Fig. 6b). In parallel, no significant maturation of collagen is observed during the mineralization (Fig. 6c) and similar results are obtained from the ratio of proline to hydroxyproline peak intensity (851:873 cm^−1^) (data not shown).

To assess the quality of the mineral deposit [25, 32, 33], the MMR was calculated from the phosphate-to-Amide I, carbonate-to-Amide I and phosphate-to-phenylalanine ratios, as illustrated in Fig. 6d–f. The Hap-to-phenylalanine ratio (958:1005 cm^−1^) increases significantly from day 3 to 14 for cells treated with Pi and from day 7 to 14 for cells treated with βG (Fig. 6d). The phosphate-to-Amide I and carbonate-to-Amide I ratios tend to slightly increase during the mineralization (Fig. 6e, f), however further experiments are needed to clarify the process. It is evident that as the calcification forms, the relative concentration of the organic matrix (proteins) is reduced.

**Fig. 7:**
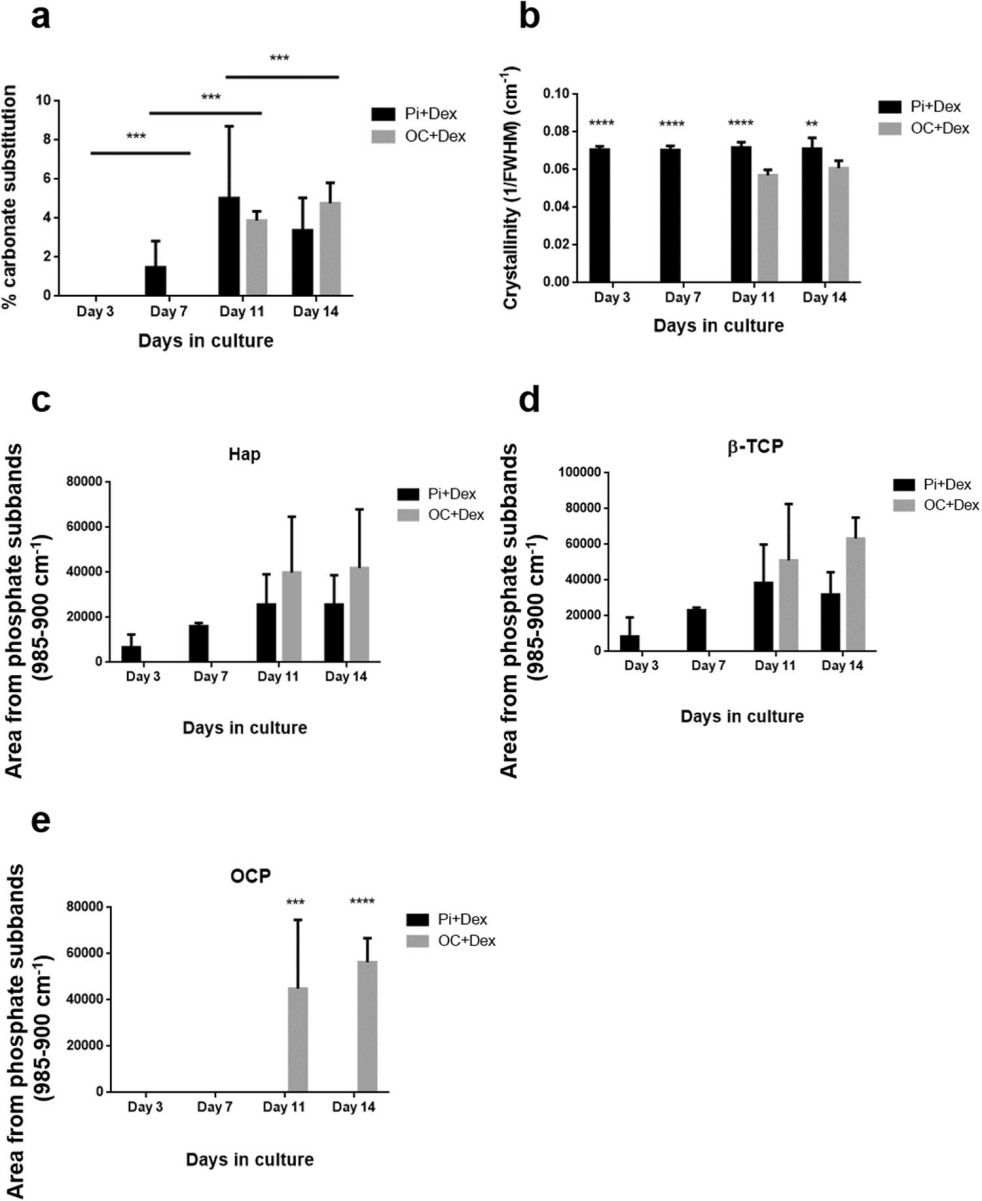
Assessment of the Hap crystallinity and combination of other minerals. **a** % carbonate substitution derived from the C:P ratio (1070/960 cm^−1^ peak intensity) for the different treatments (OC + Dex and Pi + Dex) and different days of mineralization. Two-way ANOVA test: ***P = 0.0005. **b** Hap crystallinity based on the reciprocal of the FWHM of the 960 cm^−1^ peak after 3, 7 11, and 14 days of mineralization and for both Pi and βG treatments. Two-way ANOVA test: ****P < 0.0001 and **P < 0.0001. Different calcium phosphate phases in breast cancer cell mineral deposits are found: **c** cHap, **d** OCP, and **e** β-TCP. Two-way ANOVA test: *P = 0.0215, ***P = 0.0004, and ****P < 0.0001. All data are presented as mean ± SD.

### Mineral standards as a model for microcalcifications

Mineralization is a complex mechanism which is not defined as a linear process, but rather as composed of different phases [11, 18, 20]. The timeline and stages of mineralization in breast microcalcifications are still unknown. In this study, a spectroscopic analysis of mineral standards was performed to evaluate the composition of calcium deposits in cell culture.

A series of mineral standards, namely cHap, ACP, β-TCP, and OCP, which are considered as potential precursor phases, were analyzed using Raman micro-spectroscopy. The number and position of the CO_3_^2−^ and PO_4_^3−^ peaks (Supplementary Fig. 2) were characterized based on previously published data. Supplementary Fig. 2a shows an example of Raman spectra from Hap containing different percentages of CO_3_^2−^ substitution in its lattice. For instance, the peak at 1072 cm^−1^ was assigned to the ν_1_ CO_3_^2−^ band, whilst all the other peaks are related to different vibrational modes of the PO_4_^3−^ ion. The ν_1_ CO_3_^2−^ band is well-defined and appears to increase proportionally with increasing amount of CO_3_^2−^ substitution. The PO_4_^3−^ band at 961 cm^−1^ is the most well-known and used in several studies due to its strong contribution in spectra. For instance, the area or the full-width-at-half-maximum (FWHM) of this peak is often used in the calculation of Hap crystallinity or carbonate-to-phosphate (C:P) ratio in biological samples. Several studies have also highlighted that, based on this peak, it is possible to discriminate between type A and type B CO_3_^2−^ ion substitutions [14, 22]. The ACP spectrum (Supplementary Fig. 2b) exhibits five peaks: ν_1_ PO_4_^3−^ at 955 cm^−1^, with an upshift of 4 cm^−1^ compared to the literature [34, 35], ν_3_ PO_4_^3−^ at 1034 and 1077 cm^−1^ (instead of a single peak at around 1050 cm^−1^) [34], ν_4_ PO_4_^3−^ at 588 cm^−1^ and ν_2_ PO_4_^3−^ at 427 cm^−1^, as expected. The shift and additional peaks could be due to the sample preparation, which greatly depends on temperature and time of preparation. The spectrum of β-TCP (Supplementary Fig. 2c) presents nine peaks (1088, 1015, 969, 949, 630, 548, 476, 438, and 407 cm^−1^) [34, 36], whilst the OCP spectrum (Supplementary Fig. 2d) exhibits eight peaks (1077, 1046, 1011, 958, 874, 609, 590, 427 cm^−1^) [34, 37, 38] corresponding to different vibrational modes of the PO_4_^3−^ ion. These peaks were expected and in line with previous publications.

In order to assess the carbonate content of the in vitro calcium deposits, the C:P ratio was calculated from the area under the phosphate and carbonate peaks (961 and 1070 cm^−1^, respectively) determined from curve fitting (Supplementary Fig. 3a–d). A calibration curve was derived to estimate the amount of carbonate in the calcium deposits as illustrated in Supplementary Fig. 3e. We derived a linear regression of the plot of C:P ratio vs. amount of carbonate substitution with a high regression coefficient (*R*^2^ = 0.98).

### Analysis of calcium deposits

In a similar way, a curve fitting analysis was applied to the phosphate and carbonate peaks of breast cancer cells. All peaks and their assignments are listed in Supplementary Table 1 [14, 22, 39]. Second derivative spectra were calculated for each sample to provide locations of the superimposed peaks composing the larger peak of interest and compared with the curve fitting results (Supplementary Fig. 4a, b). Typical results of curve fitting analysis applied to the spectra of cells treated with βG (OC + Dex) at day 14 of mineralization are illustrated in Supplementary Fig. 4c, e and for cells treated with Pi in Supplementary Fig. 4d, f. All sub-bands of phosphate and carbonate peaks are line with the literature [14, 22, 39] and exhibit a shift of the phosphate peak at 957 cm^−1^ for cells cultured with OC medium as previously observed.

The amount of carbonate ion substitution in each mineral deposit was determined by comparing their C:P ratio with that found in the calibration (see above). This calculation was performed for cells treated with Pi or βG at day 3, 7, 11, and 14 of mineralization (Fig. 7). Figure 7a shows an increase of carbonate content in Hap lattice for cells treated with βG during the mineralization process. In fact, the carbonate content of cells after 11 days was found to be around 3.5 wt.%, and increases to 4.7 wt.% after 14 days. While the mineralization of cells treated with Pi starts after 3 days of initiation, the incorporation of carbonate into the Hap crystal (at least to a greater level than the detection limit) appears after 7 days of mineralization, varying between 1.8 and 2.7 wt.%, and increases up to between 2 and 9.5 wt.% at day 11. Then it seems to decrease after 14 days, with a drop to between 2.3 and 5 wt.%. The variations of carbonate content in microcalcifications observed among the three replicates may be due to uncertainties in the curve fit analysis, poor spectral quality or possible differences in cHap crystal growth. Obtaining information on the crystal size for both conditions should allow the different processes of Hap crystal growth to be identified.

In fact, several studies suggest that the crystallinity or the size of Hap crystal can be related to the reciprocal of the FWHM of the ν_1_ PO_4_^3−^ peak at 960 cm^−1^ [25, 40]. Based on the results obtained from this curve fit analysis, the quantity 1/FWHM (phosphate) was calculated and the results were reported in Fig. 7b. The rate of Hap crystal formation in the case of Pi treatment is higher than for βG treatment, which is not surprising considering the mechanism of phosphate uptake by cells. In fact, βG must be hydrolyzed by the alkaline phosphatase enzyme to release a free phosphate at the surface of the cell [21, 41–43]; then, phosphate is absorbed by the cell via transporter channels. In the case of a medium supplemented with Pi, this compound is directly absorbed by the cell, enabling it to induce microcalcifications. In this context, Fig. 7b shows a stable size of Hap for days 3–14 of mineralization when cells are treated with Pi. Cells treated with βG develop a smaller crystal than cells treated with Pi.

Legeros et al. have suggested that the incorporation of ion substitution such as Mg^2+^ or CO_3_^2−^ ions in the Hap lattice can lead to a decrease in crystal size and an increase in solubility [11]. Figure 7a shows that calcium deposits released from cells treated with βG have more CO_3_^2−^ incorporated into their Hap lattice, which is in line with their lower crystal size (Fig. 7b) compared with deposits seen in cells treated with Pi.

Microcalcifications are mainly described and classified by their mineral content such as calcium oxalate and cHap. The presence of mineral precursors has been investigated in several studies of bone formation, osteoblast differentiation, or mineralization of jaw periosteal cells [20, 25, 44]. The following results are focusing on the intermediate species present in our samples, based on the curve fit analysis and summarized in Fig. 7c–e. In βG culture medium, the calcium deposits appear to be made of cHap, OCP and β-TCP at day 11 and 14. For cells treated with Pi, cHap and OCP are present in the microcalcifications at day 3–14. Moreover, no β-TCP deposits are found in these microcalcifications, which is in contrast with those found in the βG culture medium. These results reveal that the calcium deposits are subtly different between osteogenic culture media. Calcium deposits are more stable in terms of crystallinity, carbonate incorporation, and higher rate of occurrence for a medium supplemented with Pi than βG culture medium. Other approaches such as those based on X-ray diffraction may be further applied to determine the elemental composition and whether the β-TCP is magnesium-substituted, as it is found in other calcifications [8, 19].

## Discussion

Raman micro-spectroscopy allowed the in vitro mineralization of breast cancer cells to be monitored. A phosphate peak at 956/960 cm^−1^ was observed, suggesting the production of phosphate species by cells during mineralization. The analysis showed that the cells decrease their proliferation to start mineralization by stopping their mitogenesis, which reflects the variations of DNA peak intensity [20]. The calculation of MMR allows to demonstrate that, in general, there is a decrease of organic matrix and increase of mineral components over time from initiation of the mineralization process.

This study showed a higher rate of mineralization (at day 3) for cells exposed to a medium supplemented in Pi rather than βG (day 11). In fact, PCA allows a discrimination of two independent pathways of mineralization with two different clusters at day 3 and 7 between control group and cells treated with βG *vs* cells treated with Pi.

This model highlights a shift of the phosphate peak position at 956 cm^−1^ for cells treated with βG, suggesting the presence of intermediate phosphate species such as β-TCP or OCP [29] which is supported by several studies of microcalcifications in breast tissues [15, 19]. Moreover, spectra of cells treated with Pi exhibit a stable Hap crystal compared to those of cells supplemented with βG. Previously, Gosling et al. using crystallographic analysis have reported changes in crystal formation for microcalcifications in breast cancer tissues due to variations in cell microenvironment [18]. For instance, in benign lesions with a neutral pH, ACP is a precursor of crystal formation, and a high carbonate level is found in mature Hap crystal. However, the pH is more acidic for invasive lesions, leading to the involvement of an OCP precursor and a lower carbonate content within the Hap lattice [18]. Our findings suggest that β-TCP may play a role as precursor of Hap formation, suggesting that the process may be more complex than previously thought. Moreover, the incorporation of carbonate or other ions (e.g. Mg^2+^) could influence the cell behavior and cancer progression [5, 45]. Further analyses are necessary to understand the full process of microcalcification maturation and to investigate if a particular phosphate species could be a predictive marker for malignancy in breast.

In parallel, the study of the spatial distribution of the mineral deposits in the breast cancer cells was investigated and show promising results for locating the microcalcifications. It seems that the mineral deposits are mainly in the cytoplasm of the cells or the edge of the cells. At this point, we cannot be sure they are released in the medium. A higher spatial resolution (~0.5 µm) is necessary to assess precisely the location of these mineral deposits.

However, this model will be useful to correlate spectra from in vitro calcium deposits at different days of mineralization and compare them, in terms of spectral characteristics, with those from breast tissue sections at different stage of the pathology.

Our mineralization model is based on a 2D cell culture to recreate the development of microcalcifications in mammary glands; cells are grown on plastic dishes in 2D, which does not fully replicate in vivo conditions. Vidavsky et al. have developed a 3D model made of breast cell spheroids containing microcalcifications, by adding calcium and magnesium at similar concentrations as their blood level counterparts without osteogenic agents [46]. Measurements of the spheroids using Raman micro-spectroscopy at different times of maturation could be used to directly mimic the natural formation of microcalcifications within breast tissues.

A further limitation of this study is that cells were grown on CaF_2_ slides to facilitate Raman micro-spectroscopy. MDA-MB-231 cell growth characteristics on the CaF_2_ surface differed to their growth on regular tissue culture plastic and a loss of cells was observed in the longer time points for the Pi treated groups which is not observed when grown on plastic under similar conditions.

This study aimed to tease out the different mineral phases found in an in vitro model of mammary microcalcifications with increasing maturity. Vibrational spectroscopy has shown its potential for the investigation of microcalcifications by giving information about their chemical composition and spatial distribution within breast tissues. This work also contributes to understanding the mineralization process in pathological conditions, demonstrating that the tissue physiology can change the mineral composition, even in identical cells.

## Supplementary information

Supplementary Material

## Data Availability

The datasets used and/or analyzed during the current study are available from the corresponding author on reasonable request.
