# Supplementary material for: A time-course Raman spectroscopic analysis of spontaneous in vitro microcalcifications in a breast cancer cell line
Source: Lab Invest. 2021 Jun 11;101(9):1267–80. doi: 10.1038/s41374-021-00619-0 (PMC8367820; doi:10.1038/s41374-021-00619-0)
Supplement: Supplementary file 1 — Supplementary Material [file 41374_2021_619_MOESM1_ESM.pdf]

## SUPPLEMENTARY INFORMATION

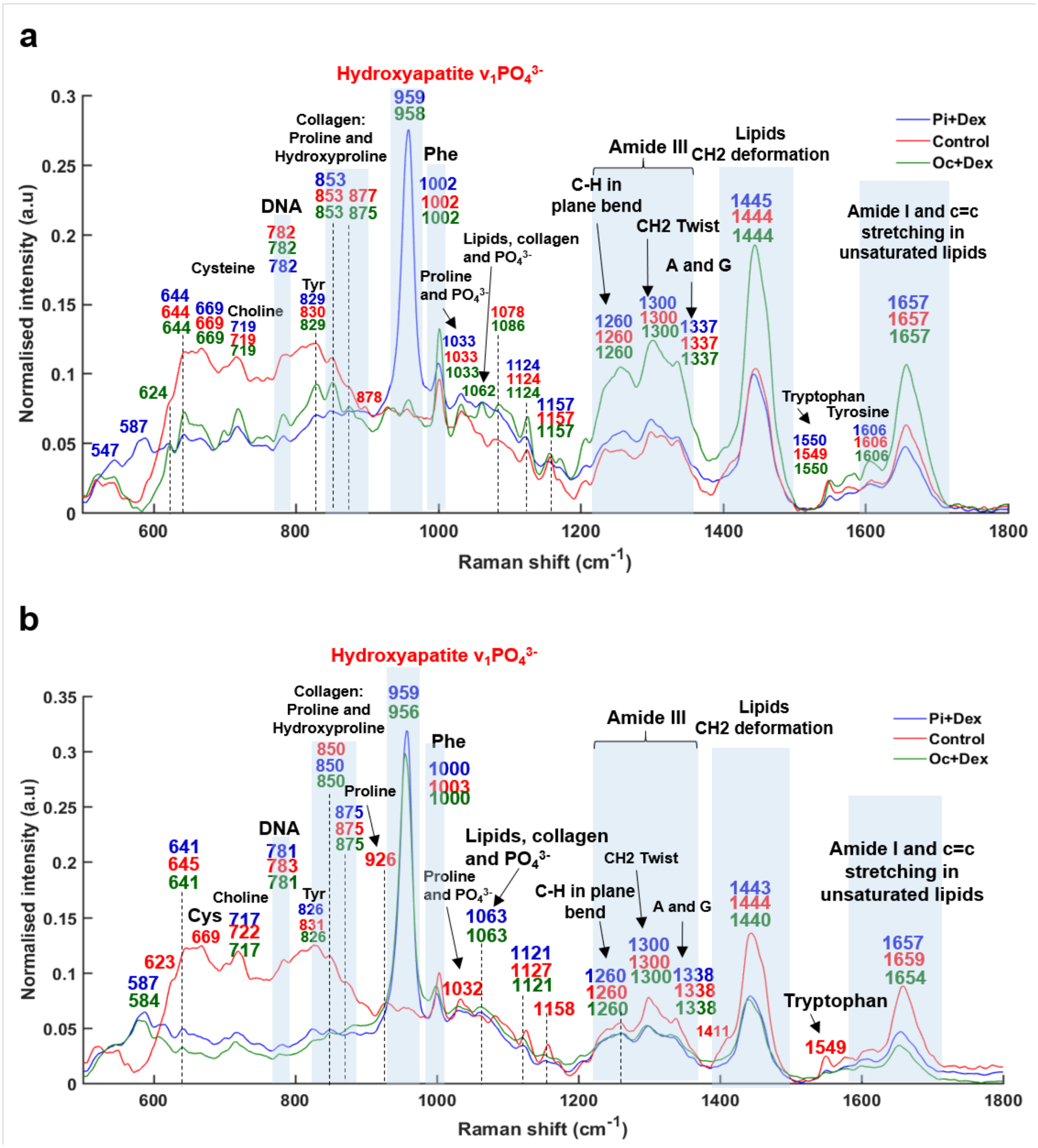


**Supplementary Figure 1.** Raman spectra acquired from mineral deposits in cell culture after 7 days (a) and 14 days (b) of mineralisation. Pi: inorganic phosphate, OC: osteogenic cocktail, Dex: Dexamethasone, Phe: Phenylalanine, Cys: Cysteine, Tyr: Tyrosine. Cells were treated with Pi+dex (blue spectrum) and OC+Dex (or βG) (green spectrum). Non-treated cells are considered as control (red spectrum). Each spectrum is an average of 40 spectra for each condition.

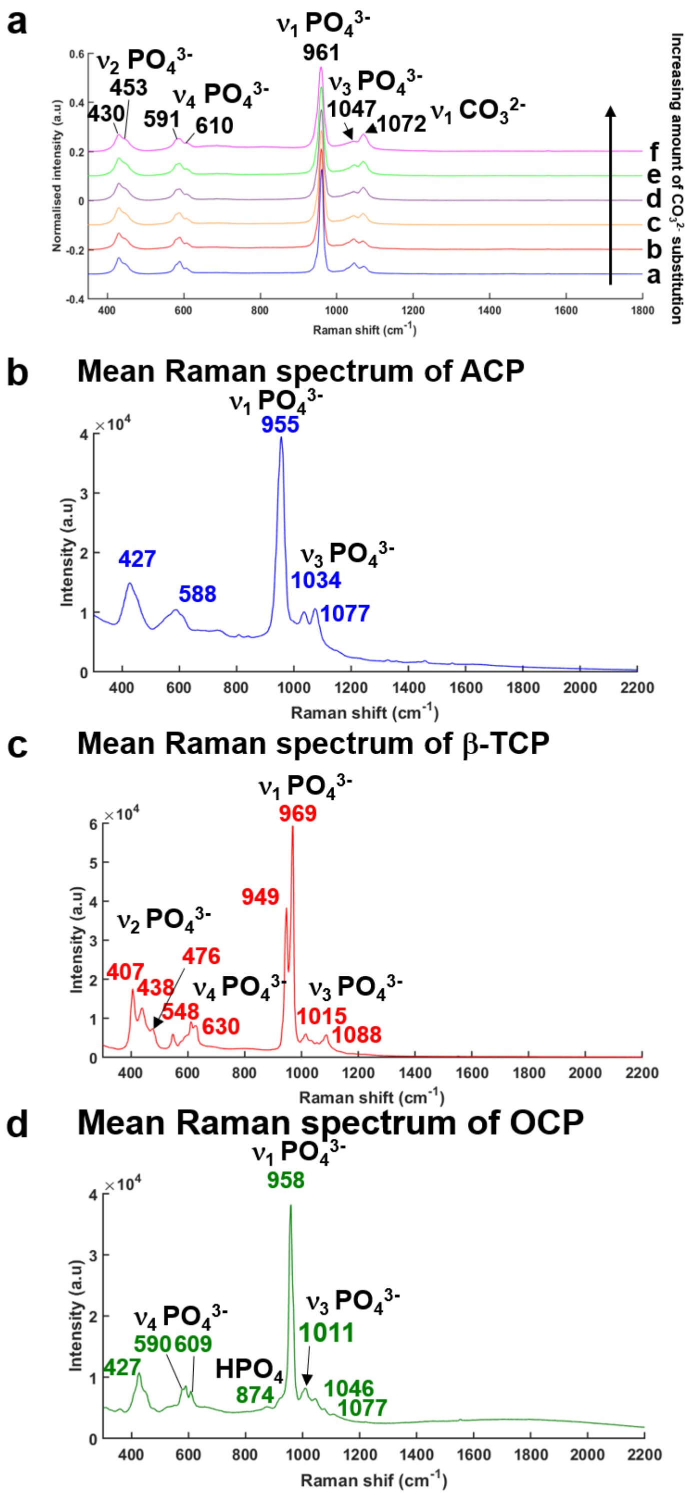


**Supplementary Figure 2.** Micro-Raman spectra of (a) cHap with different percentages of carbonate substitution, (b) amorphous calcium phosphate (ACP), (c) β-tricalcium phosphate (β-TCP) and (d) octacalcium phosphate (OCP).

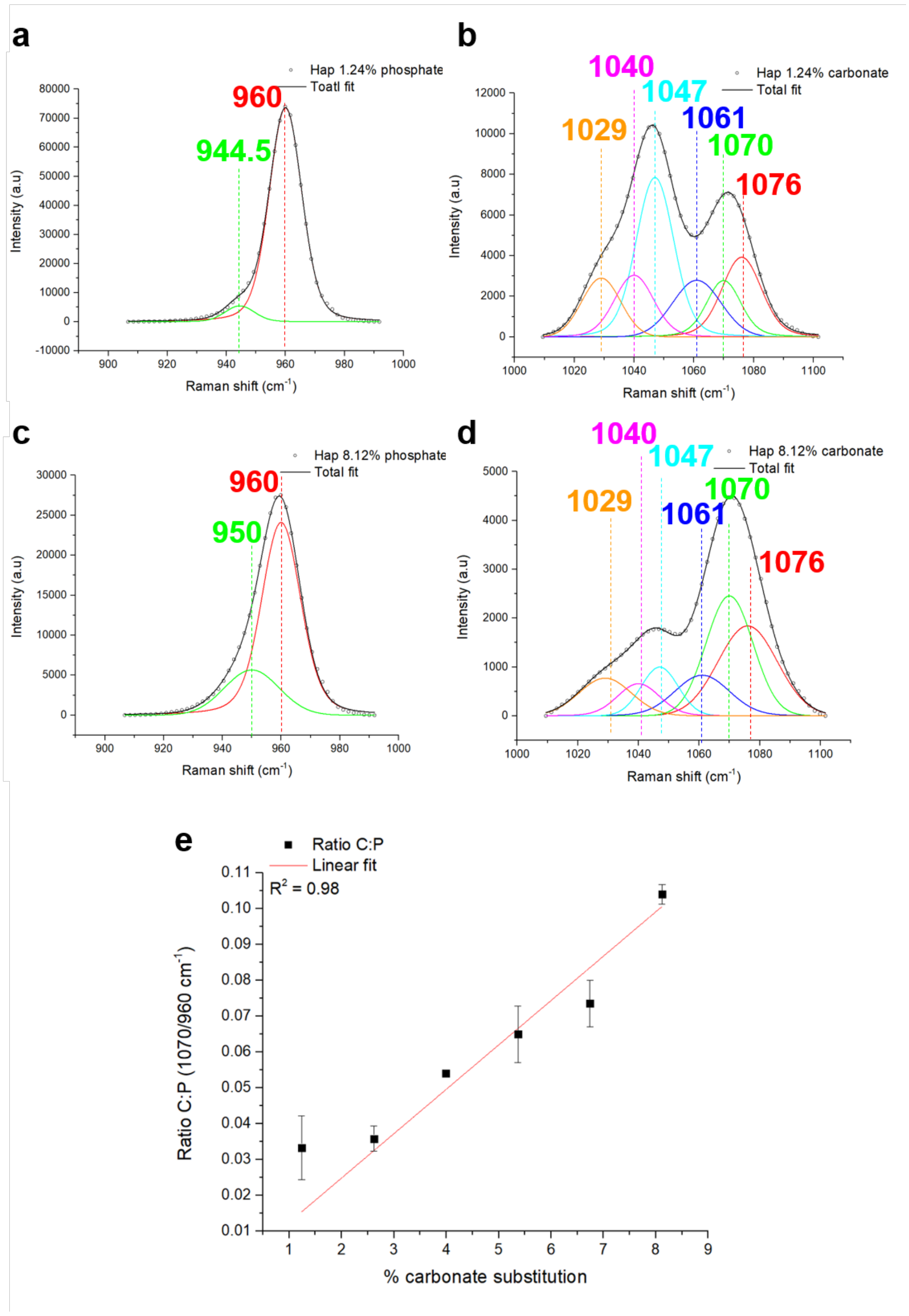


**Supplementary Figure 3.** Results of curve fitting applied to cHap standards containing (upper panels) 1.24 and (lower panels) 8.12 wt.% $CO_3^{2-}$ substitution in the (a) phosphate and (b) carbonate region ($R^2 > 0.999$). (e) Linear regression of the plot of C:P ratio vs. % $CO_3^{2-}$ substitution in cHap pellet samples. Data were obtained from Raman spectra as the ratio of the peak area at 1070 $cm^{-1}$ to 960 $cm^{-1}$. Two replicates were performed in those experiments. Values are mean ± SD. Regression equation: $y = 1.239 \cdot 10^{-2}$ x; $R^2 = 0.98$.

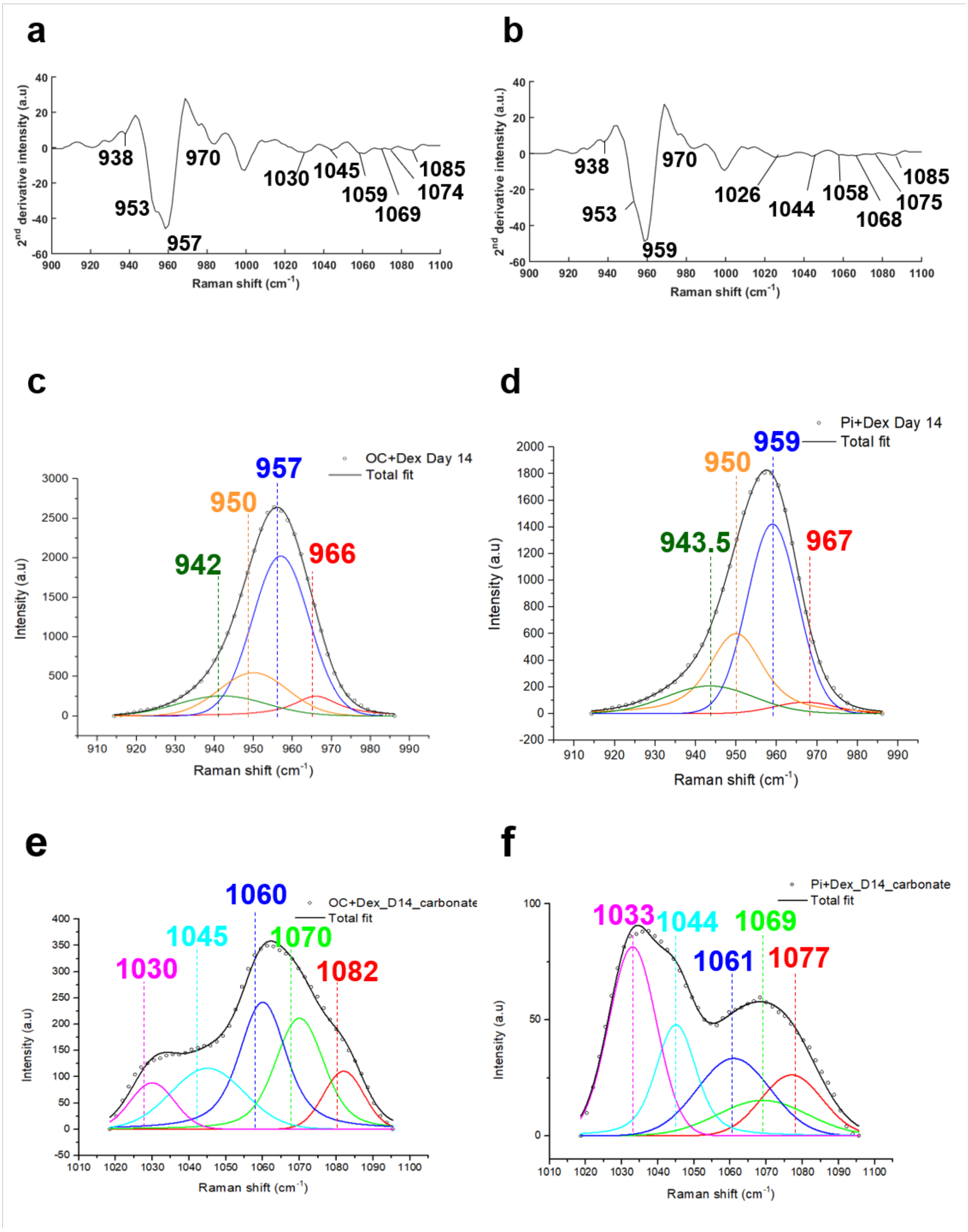


**Supplementary Figure 4.** Typical results of curve fit analysis applied to Raman spectra of breast cancer cells after 14 days of mineralisation. Second derivative spectra of cells treated with βG and Pi (a and b, respectively). Typical curve fitting results for the (c,d) phosphate and (e,f) carbonate bands of cells treated with βG and Pi. For each case, $R^2 > 0.99$.

**Supplementary Table 1.** Peak assignment for Raman bands of mineral standards.

| Peak position ($cm^{-1}$) | | | | | | Assignment | References |
|---|---|---|---|---|---|---|---|
| **• cHap standards** | | | | | | | |
| 1.24 | 2.92 | 4.43 | 5.24 | 7.52 | 8.12 wt.% | | |
| 945 | 945 | 945 | 945 | 948 | 950 | $\nu_3$ $PO_4^{3-}$ | |
| 960 | 960 | 960 | 960 | 960 | 960 | $\nu_1$ $PO_4^{3-}$ | |
| 1029 | 1029 | 1029 | 1029 | 1029 | 1029 | | |
| 1040 | 1040 | 1040 | 1040 | 1040 | 1040 | | G. Penel, 1998 |
| 1047 | 1047 | 1047 | 1047 | 1047 | 1047 | $\nu_3$ $PO_4^{3-}$ (1029–1076) | A. Antonakos, 2007 |
| 1061 | 1061 | 1061 | 1061 | 1070 | 1061 | | A. Awonusi, 2007 |
| 1070 | 1070 | 1070 | 1070 | 1061 | 1070 | | |
| 1076 | 1076 | 1076 | 1076 | 1076 | 1076 | | |
| | | | | | | $\nu_1$ $CO_3^{2-}$ | |

| Peak position ($cm^{-1}$) | | | Assignment | References |
|---|---|---|---|---|
| **• Other minerals** | | | | |
| ACP | β-TCP | OCP | | |
| 945 | | 945 | $\nu_3$ $PO_4^{3-}$ | |
| 958 | 957 | | | |
| | | 960 | $\nu_1$ $PO_4^{3-}$ (960–1040) | |
| | 966 | 968 | | |
| 1029 | | | | |
| 1040 | | | | |
| 1061 | | | | G. Penel, 1998 |
| 1070 | | | $\nu_3$ $PO_4^{3-}$ (1061–1076) | A. Awonusi, 2007 |
| 1076 | | | | |
| 1086 | | | $\nu_1$ $CO_3^{2-}$ | |
| | | | $\nu_3$ $PO_4^{3-}$ | |
